# Increased Outdoor PM_2.5_ Concentration Is Associated with Moderate/Severe Anemia in Children Aged 6–59 Months in Lima, Peru

**DOI:** 10.1155/2019/6127845

**Published:** 2019-07-24

**Authors:** Valeria C. Morales-Ancajima, Vilma Tapia, Bryan N. Vu, Yang Liu, Dulce E. Alarcón-Yaquetto, Gustavo F. Gonzales

**Affiliations:** ^1^Endocrinology and Reproduction Unit, Research and Development Laboratories (LID), Faculty of Sciences and Philosophy, Universidad Peruana Cayetano Heredia, Lima, Peru; ^2^Department of Biological and Physiological Sciences, Faculty of Sciences and Philosophy, Universidad Peruana Cayetano Heredia, Lima, Peru; ^3^Instituto de Investigaciones de la Altura, Universidad Peruana Cayetano Heredia, Lima, Peru; ^4^Rollins School of Public Health, Emory University, Atlanta, GA, USA; ^5^Biomedical Informatics in Global Health Unit, School of Public Health, Universidad Peruana Cayetano Heredia, Lima, Peru

## Abstract

Anemia affects 1.62 billion people worldwide. Although iron deficiency is the main cause of anemia, several other factors may explain its high prevalence. In this study, we sought to analyze the association between outdoor particulate matter PM_2.5_ levels with anemia prevalence in children aged 6–59 months residing in Lima, Peru (*n* = 139,368), one of the cities with the worst air pollution in Latin America. The study period was from 2012 to 2016. Anemia was defined according to the World Health Organization (Hb < 11 g/dL). PM_2.5_ values were estimated by a mathematical model that combined data observed from monitors, with satellite and meteorological data. PM_2.5_ was analyzed by quintiles. Multiple linear and logistic regressions were used to estimate the associations between hemoglobin concentration (beta) and anemia (odds ratio) with PM_2.5_, after adjusting by covariates. Prevalence of anemia was 39.6% (95% confidence interval (CI): 39.3–39.9). Mild anemia was observed in 30.8% of children and moderate/severe in 8.84% of children. Anemic children compared with nonanemic children are mainly males, have low body weight, higher rate of stunting, and live in an environment with high PM_2.5_ concentration. A slight decrease in hemoglobin (4Q B: −0.03, 95% CI: −0.05 to −0.02; 5Q B: −0.04, 95% CI: −0.06 to −0.01) and an increase in the probability of moderate/severe anemia (4Q OR: 1.18, 95% CI: 1.10–1.27; 5Q OR: 1.18, 95% CI: 1.08–1.29) were observed with increased exposure to PM_2.5_. We conclude that outdoor PM_2.5_ levels were significantly associated with decreased hemoglobin values and an increase in prevalence of moderate/severe anemia in children under 5 years old.

## 1. Introduction

Anemia affects 1.62 billion people worldwide [1] and is associated with numerous adverse health outcomes, including increased mortality and cognitive disorders. Developing countries account for 89% of all anemia-related disability [2].

According to the WHO, 50% of cases of anemia are due to iron deficiency, while 42% of the cases are of inflammatory origin (inflammatory anemia). Inflammatory anemia or anemia of inflammation (AI) in children aged 6–59 months is mainly related to infectious diseases particularly in less developed countries [3]. However, infections are not the only cause for inflammation. Evidence suggests that chronic diseases such as chronic kidney disease, congestive heart failure, chronic pulmonary disease, and obesity may result in AI [4, 5].

Another potential cause of inflammatory anemia is exposure to air pollutants since fine particulate matter equal or less than 2.5 microns in diameter (PM_2.5_) and NO_2_ produce systemic inflammation [6] and directly affect bone marrow [7]. From these, PM_2.5_ is one of the most toxic pollutants, and short-term exposure to PM_2.5_ causes a 2.8% increase in mortality rates with adverse effects in human health [8]. In addition, long-term exposure to PM_2.5_ is associated with cardiovascular disease and chronic obstructive pulmonary disease [9] and may cause oxidative stress-dependent inflammation in the lungs [10, 11].

Inflammation-inducible cytokines increase serum levels of hepcidin, the main regulator of iron homeostasis, which in turn will block iron absorption in the duodenum and cause iron retention in reticuloendothelial cells resulting in iron-restricted erythropoiesis. In addition, shortened erythrocyte half-life suppressed erythropoietin response to anemia and inhibition of erythroid cell differentiation by inflammatory mediators, further contributing to AI [3]. Oxidative stress has been considered as a cause of eryptosis or shortening of erythrocyte half-life [12].

An emerging body of evidence has associated exposure to air pollution with anemia in an older population, with C-reactive protein, an inflammatory marker acting as a mediator [11]. In addition, exposure to long-term PM_2.5_ and NO_2_ was associated with decreased cognitive function [13]. Most of these studies relating air pollution with inflammation and anemia have been performed in older people in whom it is possible to determine long-term exposure. According to different studies, all of these adverse outcomes seem to be related to moderate/severe anemia [1].

It is important to know if the effect of air pollution on hemoglobin (Hb) and anemia is also observed in other age groups and how associated it is to the severity of anemia. A study in 29 developing countries revealed that both moderate exposure and high exposure to biofuel smoke at the country level were associated with moderate/severe anemia in children (odds ratio (OR): 2.36; 95% confidence interval (95% CI): 1.28–4.36 vs OR: 2.80; 95% CI: 1.37–5.72) [14].

A strong association between biofuel use and risk of anemia and stunting in children suggests that exposure to biofuel smoke may contribute to chronic nutritional deficiencies in infants. 31% of moderate-to-severe anemia and 37% of severe stunting among children aged 6–35 months in India may be attributable to exposure to biofuel smoke [15].

Despite there are data regarding indoor air pollution and anemia, the effect of outdoor air pollution, especially PM_2.5_, on anemia in children is still unknown.

According the World Health Organization (WHO), Lima, Peru, is one of the Latin American cities with the highest mean annual PM_2.5_ values, a matter of severe public health concern [16]. In fact, PM_2.5_ levels in Lima between 2001 and 2011 averaged 50 *μ*g/m^3^. It was estimated that the excess of PM_2.5_ in Lima (above 10 *μ*g/m^3^ of the reference level) during this period, resulted in approximately 2,300 premature adult deaths from cardiorespiratory disease annually [17].

Air quality guidelines given by the WHO state that the annual mean value of PM_2.5_ concentration should be 10 *μ*g/m^3^ and 25 *μ*g/m^3^ for a 24-hour mean [18]. This threshold is not met by many countries including Peru, where the Ministry of Environment (MINAM) recommends a threshold of 25 *μ*g/m^3^ for an annual mean and 50 *μ*g/m^3^ for a 24-hour mean [19]. These different guidelines, besides creating confusion, might have effects when trying to assess the impact of air pollution on health.

For the aforementioned reasons, the present study was designed to determine the relationship between Hb and anemia with PM_2.5_ concentration in children aged 6–59 months residing in Lima, Peru. The study also aims to determine the association between anemia levels and high PM_2.5_ values using the thresholds provided by the WHO and MINAM.

## 2. Materials and Methods

### 2.1. Study Area

The metropolitan city of Lima is the capital of Peru with more than 10 million inhabitants. It is located in the central coast of the country (150 m.a.s.l.), next to the Pacific Ocean. Lima is divided into 43 districts, which cover 2,672.28 km^2^ of the land; those include the actual city (825.88 km^2^) and the city outskirts (1,846.40 km^2^). For this study, districts of Lima have been divided into 5 zones: North Lima, Center Lima, South Lima, East Lima, and West Lima (which include the constitutional region of Callao). Center Lima is considered more economically developed than the rest of the zones, but the entire city is highly urbanized. The population density of Lima by district can be seen in Supplementary Figure 1(a).

### 2.2. Study Population

The study analyzed the Nutritional Status Information System (SIEN in Spanish), a database provided by the National Center of Feeding and Nutrition (CENAN in Spanish), an institution belonging to the National Institutes of Health in Peru. Data analyzed in the current study included 139,368 infants and children aged 6 to 59 months with permanent residence on the districts that comprise the metropolitan city of Lima. Distribution of the sample by district can be seen in Supplementary Figure 1(b). 4,208 registries lacked hemoglobin (Hb) data or had implausible Hb values < 3 g/dL (*n* = 28) or >30 g/dL (*n* = 64). These data were excluded from analysis. Data were collected yearly from 2012 to 2016.

### 2.3. Variables

Hemoglobin (Hb) was measured in each health center by trained personnel using the Hemocue method and is expressed in grams per deciliter (g/dL). Anemia in children aged 6 to 59 months was defined as Hb < 11 g/dL according to the WHO guidelines [20]. Anemia was categorized according to severity as mild (10–10.9 g/dL), moderate (<10–7 g/dL), and severe (<7 g/dL) [21].

Daily PM_2.5_ exposure levels from 2010 to 2016 were estimated using a random forest (RF) model [22]. The RF model integrates satellite remote sensing aerosol optical depth (AOD) data with meteorological variables from chemical transport and forecast models and land use variables to predict existing ground measurements from the SENAMHI (10 stations) and Johns Hopkins (6 aggregated stations) networks. MAIAC (Multiangle Implementation of Atmospheric Correction) retrieved AOD was cross-validated with ground AOD from Arica (https://aeronet.gsfc.nasa.gov/cgi-bin/type_one_station_opera_v2_new?site=Arica&nachal=2&level=1&place_code=10), the nearest AERONET site located in Chile for 2010 to 2015, and gap-filled through a random forest model (cross validation (CV) *R* ^2^ = 0.82) [18].

Meteorological variables from WRF-Chem (Weather Research and Forecast model coupled with Chemistry) and ECMWF (European Centre for Medium-Range Weather Forecasts) were collected at 5 km^2^ and 1 km [2] spatial resolution, respectively, and interpolated to 1 km^2^ resolution through inverse-distance weighting. Land use variables such as percent urbanization, elevation, population density, and distance to road were also calculated for use in the model. The RF model to predict daily PM_2.5_ levels achieved an *R* ^2^ (and CV *R* ^2^) of 0.70 (RMSE = 5.95 *μ*g/m^3^, CV RMSE = 5.97 *μ*g/m^3^) [22].

Finally, we assigned the average of the last month prior to the measurement of hemoglobin to each child. PM_2.5_ data was estimated from each participant's district of residence.

Other variables were age (expressed in months) and stunting (defined as height for age Z-score (HAZ) < −2.0). Lima area includes the 43 districts grouped as north (8 districts), center (15 districts), south (12 districts), east (8 districts), and west (1 district, Callao) (Supplementary Figure 2). The poverty index as defined by the Peruvian Institute of Statistics and Informatics (INEI) [20]. In brief, their prediction model uses the logarithm of the per capita income along with data of the National Household Survey that includes variables such as educational characteristics, economically active population, availability of electric lighting, type of water supply, hygienic services, material of the walls and floors of the house, possession of electronic equipment, and telecommunication services. This index was used as a continuous variable. The variables year and month correspond to the date of the measurement of hemoglobin. Season was defined as cold and hot months.

This study was approved by the IRB at the Universidad Peruana Cayetano Heredia (SIDISI 103700).

### 2.4. Statistical Analysis

Software STATA 14.0 (StataCorp, Texas, USA) was used for statistical analysis and QGis 2.18.16 Las Palmas (http://www.qgis.org) was used for map construction. Data are presented as mean values ± standard deviations for continuous variables and as percentages for categorical variables. For statistical analysis, anemia was further categorized as mild and moderate/severe since cases of severe anemia were scarce. PM_2.5_ concentration does not have normal distribution; therefore, nonparametric tests were used to compare PM_2.5_ and anemia in the different zones of Lima. For categorical variables such as anemia and stunting, the chi-square test was used to compare them between the five zones of Lima. Differences between normally distributed variables among the five zones of Lima were assessed with ANOVA test. Those variables that were not normally distributed were assessed with nonparametric Wilcoxon test.

Multiple linear regression and logistic regression models were used to associate hemoglobin concentration or prevalence of anemia with PM_2.5_ concentration. In addition, severity of anemia was evaluated by logistic regression with two models, one for mild anemia and the other for moderate/severe anemia. Both crude and adjusted models were run. Adjustment was made with age, gender, stunting, year, month, Lima zone, poverty, and season. First, we analyzed PM_2.5_ as a continuous variable, then PM_2.5_ values were categorized in quintiles: 1Q: 17.39 *μ*g/m^3^ (13.62–18.22); 2Q: 19.18 *μ*g/m^3^ (18.23–20.25); 3Q: 21.89 *μ*g/m^3^ (20.26–24.89); 4Q: 26.87 *μ*g/m^3^ (24.97–28.83); and 5Q: 32.69 *μ*g/m^3^ (28.85–51.0). We additionally generated a new variable with values recommended by the WHO (25 *μ*g/m^3^) and MINAM (50 *μ*g/m^3^) as cut-off of permissible PM_2.5_ values in order to compare the risk between both thresholds.

To assess if a certain age group is more vulnerable to the exposure, we further stratified by age from 6 to 11, 12 to 35, and 36 to 59 months. This categorized variable has been included in all statistical models. A *p* value <0.05 was considered as significant.

## 3. Results and Discussion

### 3.1. Results

Characteristics of the study population are shown in Table 1. From the total population (*n* = 139,368 children), 55,216 (39.6%) children had anemia (Hb < 11 g/dL). Anemic children compared to nonanemic children are mainly young children, male, with low body weight, higher rate of stunting, and live in an environment with high PM_2.5_ concentration. Mild anemia was observed in 30% of the population, while severe/moderate anemia was observed in 8.8% of the population.

In Table 2, data related to the five zones of Lima are shown. Here, the 43 districts are included. The east sector of Lima has the lowest hemoglobin concentration (11.09 ± 1.05 g/dL, mean ± SD), the highest prevalence of anemia (41.3%), and the highest concentration of PM_2.5_ (31.6 *μ*g/m^3^ ± 5.6). The highest concentration of Hb was observed in the West zone, and it was associated with the lowest prevalence of anemia, lower prevalence of stunting, and lower PM_2.5_ values.

**Figure 1:**
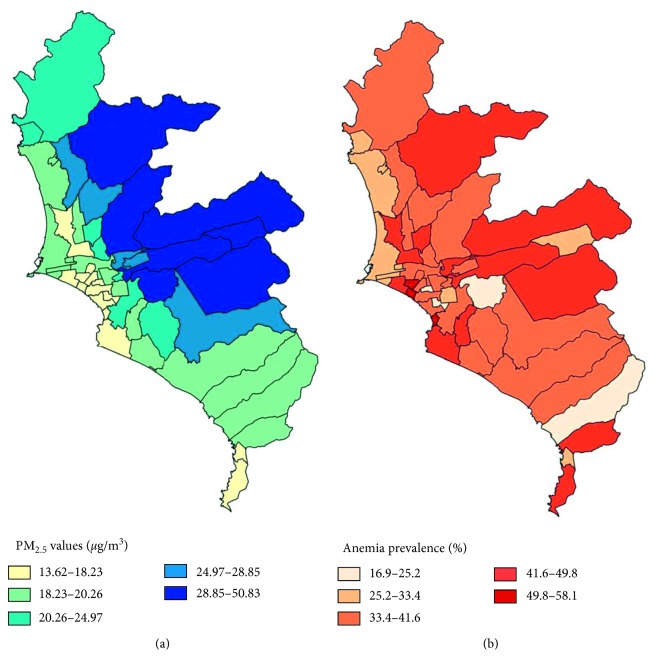
(a) Average PM_2.5_ concentration by district. 15 districts fall in the 1st quintile (1Q), 11 in the 2Q, 6 in the 3Q, 5 in the 4Q, and 7 districts in the 5Q. (b) Anemia prevalence in children aged 6–59 months during the period of the study (2012–2016). 4 districts fall in the 1Q, 6 in the 2Q, 19 in the 3Q, 12 in the 4Q, and 3 districts in the final quintile.

Figure 1(a) shows the different concentrations of PM_2.5_ in the districts of metropolitan Lima from 2012 to 2016. The lighter blue tone corresponds to lower concentration of PM_2.5_ (13.6–18.23), and the darkest tone corresponds to the highest concentration of PM_2.5_ (28.85–50.83).  Figure 1(b) shows the prevalence of anemia by district during the same period. The lighter tone of red corresponds to the lower anemia prevalence (16.9–25.2%), and the darkest color corresponds to the highest (49.8–58.1%). Anemia prevalence above 25% is observed in almost all districts.

The linear regression analysis between quintiles of PM_2.5_ concentration and hemoglobin showed a decline in Hb concentration with increasing PM_2.5_. Interestingly, exposure to values of the quintile 4 (24.97–28.84 *μ*g/m^3^) resulted in a significant decrease in hemoglobin levels (Table 3). Hemoglobin increases with age; is higher in female than male children, higher in cold seasons, and lower in children with stunting and in those with high poverty index; and increases over time from 2014 onwards.

When magnitude of anemia was assessed, the effect of exposure to high concentrations of PM_2.5_ was seen in moderate/severe anemia. After adjusting for several variables, the OR for moderate/severe anemia was significantly higher in quintiles 2, 3, 4, and 5, while the OR for mild anemia was not different among PM_2.5_ quintiles (Table 4). Quintile 2 includes values of PM_2.5_ below the threshold recommended by the WHO (PM_2.5_ = 25 *μ*g/m^3^).

We analyzed the thresholds used by the WHO and MINAM as cut-off point to define PM_2.5_ permissible values and their association with anemia. When using the threshold recommended by the WHO (PM_2.5_ = 25 *μ*g/m^3^), a reduction of hemoglobin is observed even at lower than permitted values (beta: −0.01; 95% CI: −0.030 to −0.01). With the threshold recommended by the MINAM (PM_2.5_ = 50 *μ*g/m^3^), a greater decline of hemoglobin concentration was observed (beta: −0.39; 95% CI: −0.595 to −0.1965).

Mild anemia and severe/moderate anemia have been evaluated at the different air quality cut-off points. When using the threshold recommended by the WHO, PM_2.5_ was associated with moderate/severe anemia (OR: 1.08; 95% CI: 1.02–1.14). However, when using MINAM reference values, PM_2.5_ over the threshold were associated with both moderate/severe anemia (OR: 2.83; 95% CI: 1.39–5.75) and mild anemia (OR: 2.76; 95% CI: 1.72–4.41).

After adjusting for confounding variables, such as age, gender, stunting, season, poverty, month, year, and Lima zone, PM_2.5_ was negatively associated with hemoglobin levels at 6–35 months of age (coefficient of regression ± standard error: 0.0015 ± 0.0007; 95% CI: −0.029 to −0.0001; *p*=0.03). This was not observed in the group of 36–59 months (*p*=0.597).

### 3.2. Discussion

This study assessed outdoor PM_2.5_ exposure and its association with anemia prevalence in children aged 6 to 59 months residents in Lima, Peru, one of the cities with the worst air pollution in Latin America. The anemia rates shown in these studies are high and are in line with official statistics for metropolitan Lima [23]. Anemia is one of Peru's major public health problems. Our findings show that exposure to increasing concentrations of PM_2.5_ is associated with a decrease in hemoglobin levels. This, in turn, increases the prevalence of anemia. For instance, when people are exposed to concentrations between 25 *μ*g/m^3^ (WHO reference value) and 50 *μ*g/m^3^ (MINAM reference value), a decrease in hemoglobin is observed. Moreover, the higher the concentration of PM_2.5_ to which a child is exposed, the greater the reduction in hemoglobin concentration.

Peru has its own guideline thresholds for PM_2.5_ that differs from the ones recommended by the WHO. The prevalence of anemia increases at 50 *μ*g/m^3^ PM_2.5_ [19]. Also, an increase in anemia prevalence is observed at concentrations between the referential air quality values of PM_2.5_ (25–50 *μ*g/m^3^), meaning that particulate matter at higher concentrations is an important health problem for populations exposed to this pollutant.

Furthermore, our results report an association with moderate/severe anemia, even with PM_2.5_ values below the threshold recommended by the WHO. Most of the children with anemia have mild anemia [1]. The group of mild anemic may be worsened in the presence of air pollution (PM_2.5_) moving anemia from mild to moderate/severe. Our hypothesis is based on the fact that threshold of PM_2.5_ recommended by the WHO and MINAM was associated to moderate/severe anemia.

As evidenced by the results in this study, children exposed to high concentrations of PM_2.5_ hold a greater risk of a decrease in hemoglobin values and an increase in anemia prevalence. With MINAM's referential daily value of 50 *μ*g/m^3^, children are at an even higher risk for moderate/severe anemia. Data from the present study reveal that even values below the threshold recommended by the WHO are associated with anemia in children. Guidelines to define permissible values of daily PM_2.5_ concentration should be revised. In Lima, the main sources of outdoor air pollution are automotive fleet and industrial activities [24]. A recent study showed that a change of policy regarding traffic regulation of one of Lima's main avenues, significantly decreased PM_2.5_ values [25], meaning that similar changes may have an impact improving air quality.

In Peru, children begin preschool after turning 3 years old. Before this age, they mainly stay at their homes, so assessing the exposure with the district of residence gives a good estimate of how permanently exposed these children are to pollutants. Furthermore, children tend to attend preschool institutions close to their homes at the same district, so estimating exposure based on the district of residence is also applicable for the older age group.

The plausibility of our findings is that air pollutants generate an inflammatory process in the human body [26, 27]. PM_2.5_ has been shown to increase systemic inflammation [28] and produce chronic inflammation [29]. These systemic processes potentially contribute to the association with low hemoglobin levels and an increase in prevalence of anemia as observed in the present study. Poursafa et. al. showed that increased levels of PM_10_ were associated with a reduction of hemoglobin concentration and red blood cell counts but increased white blood cell counts suggesting a direct association with inflammation and an inverse relationship with hemoglobin levels [30]. Furthermore, Honda et al. (2017) showed that an increased exposure to PM_2.5_ was associated with a decrease of 0.81 g/dL of hemoglobin in an elderly population [11]. Other volatile pollutants have also been linked to a decrease in Hb and other hematological markers with an increase in proinflammatory cytokines [31].

Particulate matter, as any other pollutant, generates an inflammatory response in the human body [32–35]. These effects are almost immediate since it has been shown that after a 1- to 3-day exposure to PM_2.5_, C-reactive protein levels, an inflammatory marker, increase [36]. Therefore, children under 5 years represent a good model to assess the effects of PM_2.5_ exposure and anemia.

Hepcidin, the hepatic peptide hormone in charge of controlling iron homeostasis, and ferroportin, a transmembrane receptor which exports iron into blood plasma, control the main routes of iron transport and availability and determine total body iron content [37]. Inflammation generated by PM_2.5_ increases serum hepcidin levels, degrading ferroportin as a host defense mechanism [38]. This mechanism is induced by proinflammatory cytokines that enhance hepcidin levels. This results in an increased internalization and degradation of ferroportin followed by cellular iron retention, ultimately leading to insufficient iron bioavailability. This iron deficiency can lead to an inflammatory anemia [39]. Another explanation for the link between outdoor air pollution and anemia is hemolysis or the destruction of red blood cells. This event can be triggered by the mineral particles adsorbed after airborne pollution exposure [40]. Both the cascading effect produced by PM_2.5_ on hepcidin and the hemolysis need further research.

This study has certain limitations. First, our database was obtained from the Information System of Nutritional Status (SIEN in Spanish) of the National Institutes of Health in Peru. The rates of anemia prevalence only correspond to the population which was attended at public health care centers. People attended in private health care system, which accounts for 10% of the population, and those attended by the Social Health Insurance (EsSalud in Spanish), nearly 39% [41], are not included.

This limitation may explain why some of the more residential or high-income districts have a high percentage of anemia prevalence since it does not include the populations with a high or high-average socioeconomic level that are attended at private health care centers. PM_2.5_ data have been linked to each participant's district of residence; it could not be linked to each participant's exact address. Another major limitation is that inflammatory markers have not been assessed in this study. C-reactive protein (CRP) and hepcidin levels should be measured in future studies to properly explain the mechanism by which exposure to increased PM_2.5_ increases anemia prevalence.

Even though the study is not prospective, it appears as children with mild anemia (control group) may worsen and become moderate to severe anemic when exposed to increased PM_2.5_ concentrations, particularly over 50 *μ*g/m^3^. This, however, needs to be proven in further research, as well as the higher susceptibility of younger children since PM_2.5_ is more strongly related to anemia in the younger age group.

## 4. Conclusions

Anemia is currently the major public health problem Peru is facing; this paper sheds light on other reasons that might explain the high anemia prevalence in the country. Exposure to higher than allowed (25 *μ*g/m^3^) concentrations of PM_2.5_ is associated with an increased prevalence of anemia in children aged less than five years particularly moderate/severe anemia, constituting a new risk factor to be taken into account when designing policies aiming to eradicate anemia worldwide. Since the risk of decreased hemoglobin levels is seen even at lower values than those suggested by the WHO and local regulatory agencies (MINAM), we call for a revision of current guidelines. The mechanisms by which PM_2.5_ exposure might decrease hemoglobin concentrations need to be studied further but might be related to the activation of inflammatory processes.

## Figures and Tables

**Table 1:** Characteristics of the study population, 2012–2016.

Characteristics	No anemia	Mild anemia	Moderate/severe anemia
	(*n* = 84152)	(*n* = 42894)	(*n* = 12322)
Age (months)			
6–11	31182 (37.1)^*∗*^	23799 (55.5)	7588 (61.6)
12–35	41869 (49.7)^*∗*^	17263 (40.2)	4426 (35.9)
36–59	11101 (13.2)^*∗*^	1832 (4.3)	308 (2.5)
Gender (%)			
Male	41824 (49.7)^*∗*^	21899 (51.1)	6739 (54.7)
Female	42328 (50.3)^*∗*^	20995 (48.9)	5583 (45.3)
Weight (kg) (mean ± SD)	11.1 ± 2.96^a^	9.96 ± 2.19	9.69 ± 2.01
Stunting (%)	3865 (4.59)^b^	2015 (4.70)	652 (5.29)
Hemoglobin (g/dL) (mean ± SD)	11.75 ± 0.67^a^	10.47 ± 0.30	9.39 ± 0.83
PM_2.5_ (*μ*g/m^3^) (mean ± SD)	23.8 ± 6.47^c^	23.9 ± 6.41	24.0 ± 6.41
Poverty	17.5 ± 6.9^d^	17.6 ± 6.8	17.5 ± 6.5

Values are frequencies and percentage (%) or mean ± SD. Comparison is between no anemia vs. mild and moderate/severe anemia. ^*∗*^Chi-square test: *p* < 0.001, no anemia vs mild and moderate/severe anemia. ^a^ANOVA: *p* < 0.001, no anemia vs mild and moderate/severe anemia. ^b^Chi-square test: *p*=0.001, no anemia vs moderate/severe anemia. ^c^Wilcoxon: *p*=0.001, no anemia vs mild and moderate/severe anemia. ^d^ANOVA: *p*=0.001, no anemia vs mild anemia.

**Table 2:** Characteristics of children aged between 6 and 59 months according to the district of residence in Lima, 2012–2016.

Zone (*n*)	Hb (g/dL) (mean ± SD)	Age (months) (mean ± SD)	Stunting (% (95% CI))	PM_2.5_ (*μ*g/m^3^) (mean ± SD)	Anemia (% (95% CI))
North (*n* = 50770)	11.15 ± 0.96^*∗∗*^	16.09 ± 11.1^*∗∗*^	4.96^b^ (4.77–5.15)	24.66 ± 6.07^a^	40.85^c^ (40.43–41.27)
Central (*n* = 20278)	11.21 ± 0.94	18.26 ± 12.2	4.07 (3.80–4.35)	18.76 2.11	37.60 (37.83–39.33)
South (*n* = 37007)	11.14 ± 1.01	17.11 ± 11.9	5.23 (5.00–5.46)	21.45 ± 3.87	39.70 (39.19–40.19)
East (*n* = 24957)	11.09 ± 1.03	15.75 ± 11.8	5.02 (4.75–5.30)	31.57 ± 5.63	41.07 (40.45–41.68)
West (*n* = 6356)	11.31 ± 1.14	19.21 ± 12.8	0	18.59 ± 1.17	30.07 (28.94–31.21)
Total	11.15 ± 0.99	16.76 ± 11.7	4.69 (4.57–4.79)	23.91 ± 6.42	39.62 (39.36–39.87)

Anemia: hemoglobin (Hb) < 11 g/dL. North Lima compared to others zones. ANOVA: ^*∗∗*^Hb: *p* < 0.001 North vs the rest. Age: *p* < 0.001 North vs the others. ^a^Wilcoxon test: *p* < 0.001 North vs the others. ^b^Chi-square test: stunting *p* < 0.001 North vs Central. ^c^Anemia *p* < 0.001 North vs Central, South, and West.

**Table 3:** Relationship between hemoglobin with PM_2.5_ quintiles in children aged 6 to 59 months.

PM_2.5_ (*μ*g/m^3^)	Sample (*n*)	Crude coefficient	95% CI	Adjusted coefficient	95% CI
1Q: (<18.23)	27757	1.0		1.0	
2Q: (18.23–20.25)	27865	0.005	−0.011–0.021	−0.017	−0.033–0.0001
3Q: (20.26–24.96)	27952	−0.006	−0.023–0.009	−0.006	−0.024–0.0121
4Q: (24.97–28.84)	27876	−**0.044**	−0.06–(−0.027)	−**0.026**	−0.046–(−0.005)
5Q: (28.85–51.00)	27918	−**0.042**	−0.058–(−0.025)	−**0.048**	−0.073–(−0.023)

Linear regression model adjusted by age, gender, stunting, season, poverty, months, year, and Lima zone. Bold values denote statistical significance.

**Table 4:** Logistic regression models between PM_2.5_ and mild and moderate/severe anemia.

PM_2.5_ (*μ*g/m^3^)	Sample *n*	Mild anemia (*n* = 42894)		Moderate/severe anemia (*n* = 12322)	
		Crude OR (95% CI)	Adjusted OR (95% CI)	Crude OR (95% CI)	Adjusted OR (95% CI)
1stQ (<18.23)	27757	1	1	1	1
2Q (18.23–20.25)	27865	0.97 (0.960–1.084)	1.02 (0.987–1.068)	1.02 (0.960–1.084)	**1.09** (1.025–1.166)
3Q (20.26–24.96)	27952	1.01 (0.969–1.043)	0.99 (0.949–1.034)	1.03 (0.969–1.094)	**1.09** (1.016–1.168)
4Q (24.97–28.84)	27876	**1.04** (1.009–1.086)	1.00 (0.955–1.051)	**1.10** (1.045–1.177)	**1.19** (1.097–1.281)
5Q (28.85–51.00)	27918	1.03 (0.999–1.075)	1.04 (0.984–1.105)	**1.07** (1.010–1.140)	**1.24** (1.133–1.366)

Models are adjusted by gender, stunting, season, poverty, months, year, and Lima zone. Bold values denote statistical significance.

## Data Availability

Data from this study are available upon request.

## Supplementary Materials

Supplementary Materials Supplementary Figure 1: (a) population density of Lima by district; (b) distribution of the study population by district. Supplementary Figure 2: (a) average PM_2.5_ values by Lima zone; (b) anemia prevalence (%) by Lima zone.
