# Supplementary material for: Increased Outdoor PM2.5 Concentration Is Associated with Moderate/Severe Anemia in Children Aged 6–59 Months in Lima, Peru
Source: J Environ Public Health. 2019 Jul 24;2019:6127845. doi: 10.1155/2019/6127845 (PMC6681625; doi:10.1155/2019/6127845)
Supplement: Supplementary Materials — Supplementary Figure 1: (a) population density of Lima by district; (b) distribution of the study population by district. Supplementary Figure 2: (a) average PM2.5 values by Lima zone; (b) anemia prevalence (%) by Lima zone. [file 6127845.f1.docx]

## Supplementary material


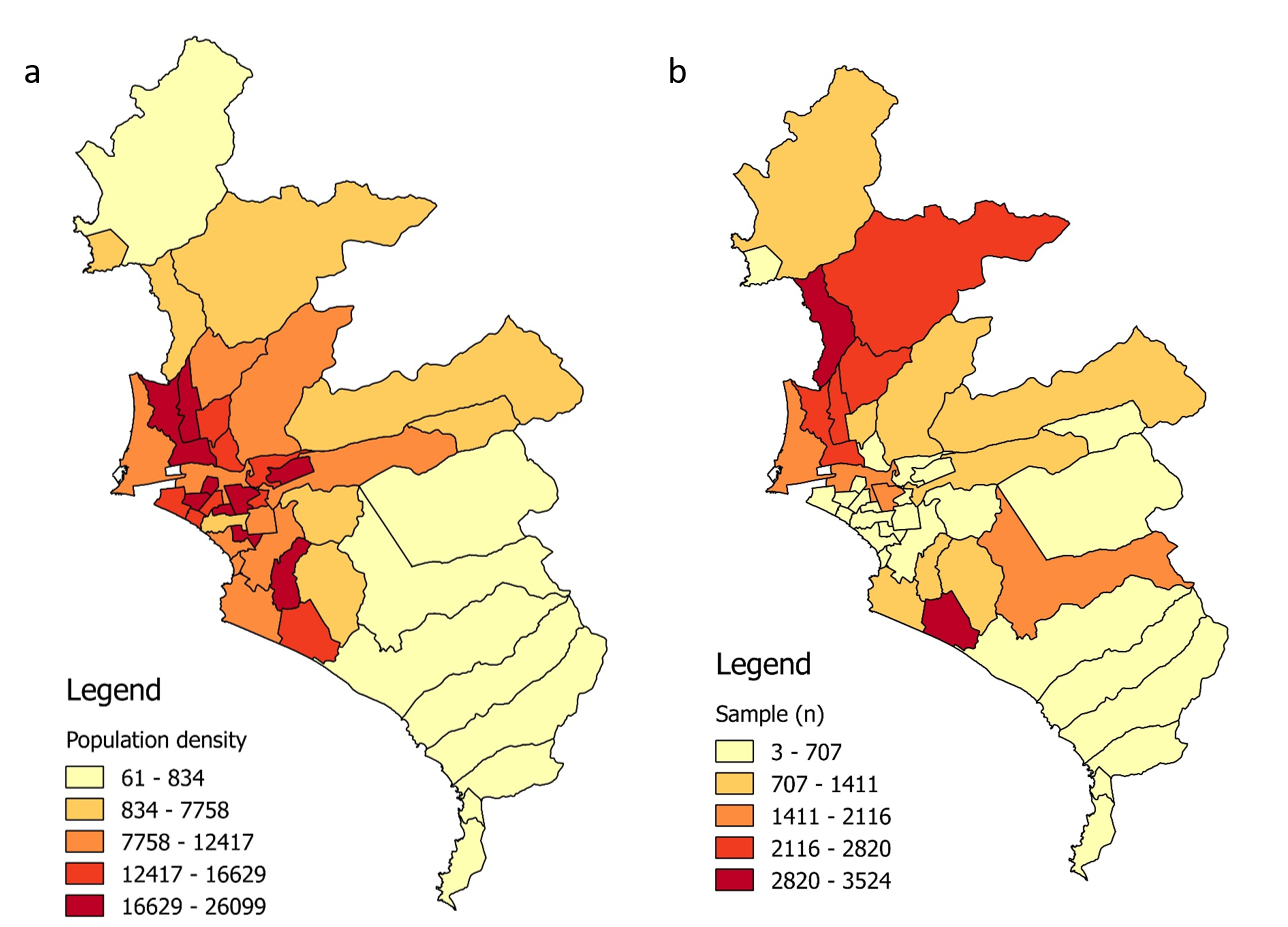


Supplementary Figure 1. (a) Population density of Lima by district (b) Distribution of the study population by district.


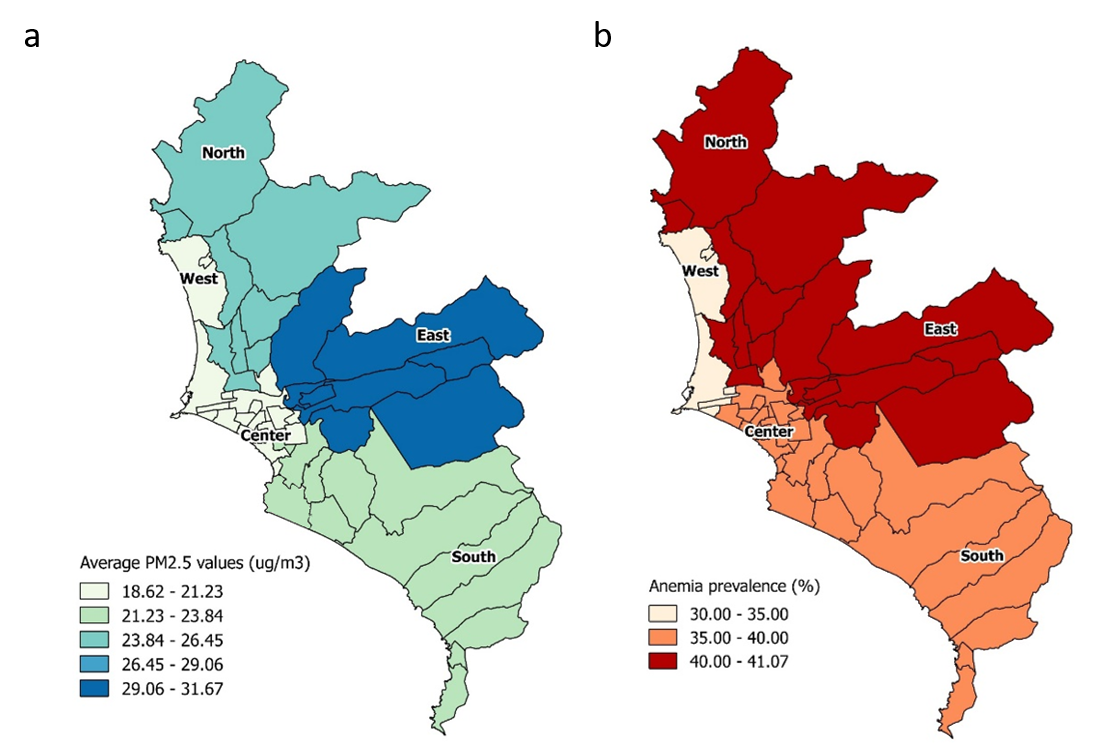
 Supplementary Figure 2. (a) Average PM_2.5_ values by Lima Zones. (b) Anemia prevalence (%) by Lima zones.
